# Effect of isolated cabg on mild-to-moderate ischemic mitral regurgitation and cardiac remodeling

**DOI:** 10.1186/1749-8090-8-S1-O214

**Published:** 2013-09-11

**Authors:** M Slavov, P Panayotov, D Panayotova, Y Peychev, V Kornovski

**Affiliations:** 1 Department of Cardiac Surgery, "St Marina" University Hospital, Varna, Bulgaria

## Background

There is no consensus regarding the need of surgical correction of mild-to-moderate ischemic mitral regurgitation (IMR) during surgical revascularization procedure. Left alone it could either improve or progress to a more severe degree and deteriorate patient’s condition, remodeling processes and quality of life.

## Methods

To evaluate the changes in structure and function of the left cardiac chambers and mitral valve apparatus after isolated surgical revascularization we perform follow-up on 69 patients subjected to CABG in the setting of mild-to-moderate IMR. Mean follow-up was 22 (6 to 52) months. Left atrial and ventricular dimensions and volumes were evaluated preoperatively and at the follow-up.

## Results

At follow-up 56 % of all survivors showed improvement in IMR grade, 32 % presented with the unchanged valve function and in only 12 % IMR grade was more severe than preoperative. Preoperative effective ejection fraction increased from 26 ± 8% to 32 ± 14% (p = 0.002) at the follow-up. Left ventricular end-systolic volume index (LVESVI) decreased from 35 ± 16 ml/m2 to 30 ± 14 ml/m2 (p = 0.001). Significant left ventricular reverse remodeling (≥ 15% reduction of LVESVI) was observed in 45 % of all survivors. In 21 % of all survivors significant left atrial reverse remodeling was present although mean left atrial volume index did not change significantly.

## Conclusions

Isolated surgical revascularization improves the valve function in ischemic mitral regurgitation. It also triggers significant left ventricular reverse remodeling in half of the patients. It remains unclear how the persisting or advancing remodeling process seen in the other half changes the prognosis of these individuals.

